# *Polygonatum cyrtonema* Hua Polysaccharides Protect BV2 Microglia Relief Oxidative Stress and Ferroptosis by Regulating NRF2/HO-1 Pathway

**DOI:** 10.3390/molecules27207088

**Published:** 2022-10-20

**Authors:** Jiayu Li, Xifan Wang, Rongrong Zhou, Fei Cheng, Xueyang Tang, Jia Lao, Linben Xu, Wei He, Dan Wan, Hongliang Zeng, Shuihan Zhang

**Affiliations:** 1 Institute of Innovation and Applied Research in Chinese Medicine, Hunan University of Chinese Medicine, Changsha 410208, China; 2 Institute of Chinese Materia Medica, Hunan Academy of Chinese Medicine, Changsha 410013, China; 3 Changsha Hospital of Traditional Chinese Medicine (Changsha Eighth Hospital), Changsha 410199, China; 4 Safety Engineering Institute, Hunan Vocational Institute of Safety Technology, Changsha 410151, China; 5 Affiliated Hospital, Hunan Academy of Chinese Medicine, Changsha 410013, China; 6 Resgreen Group International Inc., Changsha 410329, China

**Keywords:** PCP, oxidative stress, apoptosis, ferroptosis

## Abstract

Neuronal-regulated cell death (RCD) due to the accumulation of ROS within the central nervous system (CNS) is one of the crucial causes of central system diseases. Caspase-dependent apoptosis is the only form of RCD. As research progressed, several nonapoptotic cell death pathway RCDs were identified. Ferroptosis is a nonapoptotic RCD characterized by lipid peroxidation and plasma membrane damage. *Polygonatum cyrtonema* Hua. Polysaccharides (PCP) are an effective antioxidant. Based on this, the protective effect and mechanism of PCP against H_2_O_2_-induced microglial injury were investigated. Furthermore, the protective mechanism of PCP against ferroptosis in microglia was explored. Our results indicated that PCP could reduce oxidative stress-induced ROS accumulation by activating the NRF2/HO-1 signaling pathway, thus attenuating RCD in microglia. Subsequent studies have revealed that PCP alleviates ferroptosis in microglia due to protein levels of ERASTIN/RSL3 inhibitor SLC7A11/GPX4 by activating the NRF2/HO-1 signaling pathway. Therefore, we hypothesized that PCP exerts antioxidative and anti-ferroptosis effects by activating the expression of the NRF2/HO-1 pathway. This facilitates new ideas for clinically effective prevention and treatment of diseases due to accumulated reactive oxygen species in the CNS. Simultaneously, PCP has the development potential as a new drug candidate for treating CNS diseases.

## 1. Introduction

Neurodegenerative diseases, including Alzheimer’s disease (AD), Parkinson’s disease (PD), and amyotrophic lateral sclerosis (ALS), are characterized by chronic, irreversible, and progressive neuronal degenerative lesions [1]. Damage to the CNS can severely affect the patient’s quality of life and eventually lead to death [2]. The pathological changes in neurodegenerative diseases include the death of microglia, which significantly affects the environmental maintenance, damage response, and repair capacity inside the CNS [3,4]. This leads to neurodegeneration and aggravates the neurodegenerative disease. The protective mechanism of microglia on the CNS is currently unknown [2,5]. However, studies have observed its efficacy in resisting certain central neuropathies. Microglia lose their neuroprotective capacity due to reduced number and loss of function, and this loss of neuroprotection largely contributes to the development of associated neurodegenerative and neurodegenerative diseases [6,7]. On the one hand, microglia are the primary immune effector cells of the CNS and have essential functions in maintaining homeostasis in the brain and regulating neuronal surveillance [8,9]. Microglia help supports neural networks by stimulating blood vessel formation, shedding excess neurons, and enhancing cell differentiation [10,11]. On the other hand, studies have depicted that various factors could contribute to microglia death, including age, genetics, immune action, and oxidative stress. Among these, oxidative stress may have an important role [12,13].

Oxidative stress is the cellular structural damage and molecular dysfunction caused by an imbalance of reactive oxygen species (ROS) and antioxidant systems [14]. Oxidative damage is caused by the production, neutralization, or elimination of free radicals by the body through antioxidants due to an imbalance in capacity between the harmful effects [15,16]. The nuclear factor E2-related factor 2/heme oxygenase-1 (NRF2/HO-1) signal axis can resist oxidative stress by playing various physiological roles, such as antioxidation and ferroptosis [17]. Its action mechanism generally involves uncoupling KEAP1 and NRF2 during stress conditions such as ROS. NRF2 enters the nucleus to form a heterodimer with MAF protein, binding to the antioxidant response element (ARE), and regulates the activity of target genes, including superoxide dismutase (SOD), catalase (CAT), and phase II detoxifying enzymes, thus removing harmful substances such as ROS [18,19]. Interestingly, oxidative damage regulates caspase-dependent apoptosis and is associated with the underlying mechanism of ferroptosis [20,21].

Ferroptosis is a new form of cell death involving the collapse of the intracellular antioxidant defense system, the consumption of iron-dependent glutamate, and the subsequent accumulation of lipid peroxide [22]. Ferroptosis mechanisms involve inhibition of cysteine glutamate transporter (system Xc^−^), induced cysteine deficiency, glutathione (GSH) depletion in cells, GPX4 failure-mediated ferroptosis pathway, and FSP1 and CoQ10-mediated pathway [23]. On the one hand, most studies confirmed that the cysteine/GSH/GPX4 pathway only limits lipid peroxidation. Notably, when SLC7A11, a vital protein of the system Xc^−^, is inhibited, the transport of Cys is blocked, depleting intracellular GSH and causing iron-dependent ROS accumulation and cellular ferroptosis. On the other hand, the presence of high levels of intracellular Fe^2+^ is also necessary for ferroptosis [24]. It is characterized by the depletion of GSH and the decline of GPX4 activity, leading to the inability of lipid peroxides to reduce metabolism. Then, the Fenton reaction of Fe^2+^ produces a large amount of ROS, which leads to ferroptosis [25]. Our study focuses on the classical GPX4 pathway of ferroptosis; that is, when GSH is exhausted, lipid ROS will cause fatal accumulation of ferroptosis.

Currently, cholinergic, neuroprotective, antioxidants, and herbal medicines are mainly utilized to treat neurodegenerative diseases. Neuroprotective drugs, antioxidants, and Chinese herbal medicine can slow the disease process but not reverse it [26]. This also leads to significant organ function damage during long-term drug use. In addition, herbal medicine intervention helps improve the quality of the survival of patients [27]. Developing new herbal therapeutic agents targeting oxidative stress in the nervous system is much needed.

PCP is the extract of *Polygonatum cyrtonema* Hua. In traditional Chinese medicine, *Polygonatum cyrtonema* Hua is regarded to have multiple functions, including returning the spleen, lung, and kidney meridians, tonifying Qi and Yin, strengthening the spleen, moistening lungs, and benefiting the kidneys [28,29]. PCP is one of its essential pharmacological components. It has antioxidant, antiaging, antifatigue, immune enhancement, anti-inflammatory, and other pharmacological activities [30]. It has been studied as a potential ingredient against an Alzheimer’s disease for a long time. In treating nervous system diseases, PCP can significantly improve the behavior of rats with Parkinson’s syndrome and reduce the memory damage in the dementia mouse model by reducing cerebral ischemia and antioxidation. Wu Shenrong [31] observed the improvement of learning and memory of AD mice by PCP, which indicates that PCP has a specific therapeutic effect on AD. Yi Yuxing et al. [32] found that PCP elevated Aβ1-42 apoptosis of rat hippocampal cells after the intervention. The latest research indicates that *Polygonatum* polysaccharides can inhibit the lipid peroxidation induced by Fe^2+^. Here, we attempted to confirm whether PCP could protect microglia from oxidative stress-induced RCD.

## 2. Results

### 2.1. Material Basis of PCP

**Figure 1:**
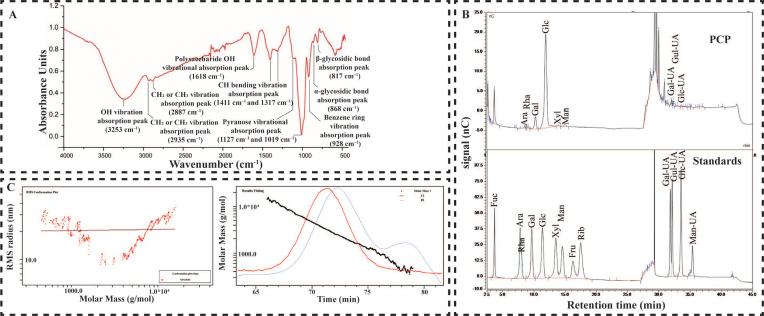
Material basis of PCP. The infrared spectrum of PCP (**A**). HAPEC ion chromatogram of PCP (**B**). HPGPC ion chromatogram of PCP (**C**).

After deproteinization with the Sevage reagent, the total sugar content of PCP purified using D101-macroporous adsorption resin was about 91.96%, and the yield became 72.20%. The PCP sample had typical absorption peaks of polysaccharides, including the CH vibration absorption peak, OH vibration absorption peak, uronic acid vibration absorption peak, and pyranose skeleton absorption peak on the sugar ring, which were detected using infrared spectroscopy. The sugar content is close to 90% (Figure 1A). After HPAEC detection, the monosaccharide composition of PCP would be: Ara 1.04%, Rha 0.31%, Gal 10.38%, Glc 80.08%, Xyl 0.26%, Man 3.12%, Gal-UA 3.98%, Gul-UA 0.31%, and Glc-UA 0.31% (Figure 1B). The peak molecular weight of PCP became 2.489 kDa (polydispersity 0.01159), and the weight-average molecular weight became 2.51 kDa (polydispersity 0.02251). Moreover, the number-average molecular weight was 1.473 kDa (polydispersity 0.0497) detected using HPGPC (Figure 1C). In this study, the extracted and purified PCP was used, and the total sugar content, functional group information, monosaccharide composition, and polysaccharide molecular weight were also examined. This facilitates a solid material basis for subsequent mechanisms.

### 2.2. PCP Attenuated H_2_O_2_-Induced Cellular Oxidative Stress and Apoptosis in Microglia

**Figure 2:**
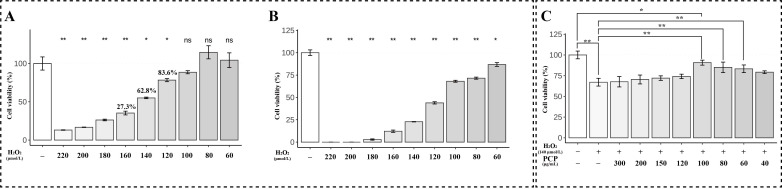
The cell viability in microglia. The cells were treated using H_2_O_2_ at different concentrations (0, 40, 60, 80, 100, 120, 140, 160, 180, and 200 μmol) for 24 h and 48 h. Then being treated with H_2_O_2_ and PCP using different concentrations (0, 40, 60, 80, 100, 120, 150, 200, and 300 μg/mL) was measured with the CCK8 assay. The cell viability of the microglia after H_2_O_2_ treatment for 24 h (**A**), 48 h (**B**), and the cell viability of microglia after utilizing H_2_O_2_ + PCP treatment (**C**). (^ns^ *p* > 0.05, * *p* < 0.05 and ** *p* < 0.01).

We simulated the oxidative stress environment with H_2_O_2_ to explore the role of PCP in microglia under oxidative stress. We used different concentrations (0, 40, 60, 80, 100, 120, 140, 160, 180, and 200 μmol) of H_2_O_2_ to intervene in microglia for 24 or 48 h in anticipation of finding suitable intervention conditions that mimic oxidative stress. When the intervention time is 24 h, we can obtain similar results to the intervention 48 h if we choose the appropriate intervention concentration, so the intervention time is finally determined to be 24 h. The final state of simulated oxidative stress of microglia is that the concentration of H_2_O_2_ was 140 μmol, and the intervention time was 24 h (Figure 2A). When this condition is intervened, microglia activity is appropriately inhibited, allowing control of the optimal economic cost versus the cost of the subsequent expedition. We used different concentrations (60, 80, and 100 μg/mL) of PCP and H_2_O_2_-treated microglia for 24 h to study the effect of PCP on H_2_O_2_-induced cytotoxicity and then observed and CCK8 analysis. The CCK8 assay revealed that microglia cell viability decreased after H_2_O_2_ treatment, but this harmful effect improved significantly in the PCP-treated groups (*p* < 0.05, Figure 2C).

**Figure 3:**
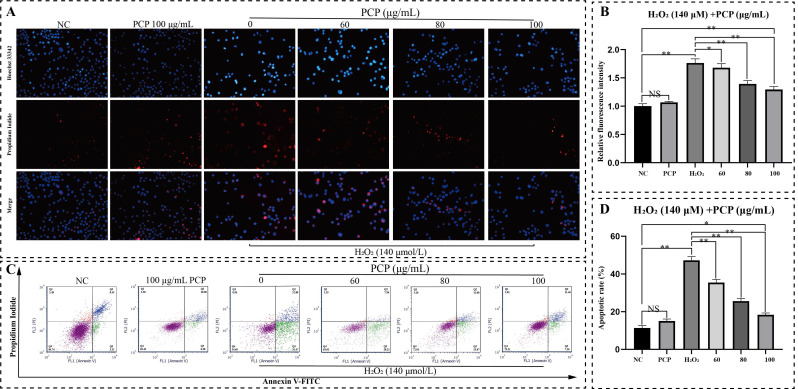
PCP attenuates H_2_O_2_-induced cytotoxicity and apoptosis in microglia. The cells were treated with H_2_O_2_ (140 μmol) and PCP at different concentrations (0, 60, 80, and 100 μg/mL) for 24 h. The NC group was set up and treated with DMEM medium as the control and PCP-treated (100 μg/mL) group. After treatment, the cell morphology was observed using Hoechst 33342/PI double staining, apoptosis was detected with flow cytometry, and the apoptosis rate was evaluated using FlowJo. Fluorescent staining results from double staining in microglia (**A**). (Q1: (AnnexinV-FITC)-/PI+; Q2: (AnnexinV+FITC)+/PI+; Q3: (AnnexinV-FITC)+/PI-; Q4: (AnnexinV-FITC)-/PI-) The relative fluorescence intensity of double staining in microglia (**B**). The apoptosis rate of the microglia (**C**). The apoptosis of the microglia (**D**). (^NS^ *p* > 0.05, * *p* < 0.05 and ** *p* < 0.01).

After double staining, most nuclei of normal living cells are uniform light blue and round. Very few dead cells were stained red, and the nuclear structure was normal. Then double staining showed a similar result: the percentage of apoptotic microglia increased significantly after H_2_O_2_ treatment. The nuclear of many microglia could be observed as bright blue and irregular in shape. There was also nuclear rupture and shrinkage. The nuclear of most cells shows red fluorescence. In contrast, PCP reduced apoptotic cells (Figure 3A). As observed in microglia, H_2_O_2_ treatment effectively enhanced the relative fluorescent intensity (*p* < 0.05, Figure 3B). However, PCP significantly scavenged intracellular relative fluorescent intensity within the treatment group (*p* < 0.05, Figure 3B). Treatment with PCP alone had no difference (*p* > 0.05, Figure 3B).

Furthermore, we also detected H_2_O_2_-induced apoptosis by FCM. The results showed that H_2_O_2_ had a noticeable apoptosis effect on the BV2 cells, but different concentrations (60, 80, and 100 μg/mL) of PCP could significantly reduce apoptosis. Moreover, there was no difference in PCP treatment alone (*p* > 0.05, Figure 3C,D). The apoptosis rate elevated after H_2_O_2_ treatment but decreased in the PCP treatment group, and the apoptosis rate altered significantly. (*p* < 0.05, Figure 3C,D).

### 2.3. PCP Upregulated BAX/Bcl2/Cleaved-Caspase3 Apoptosis Pathway in Microglia

**Figure 4:**
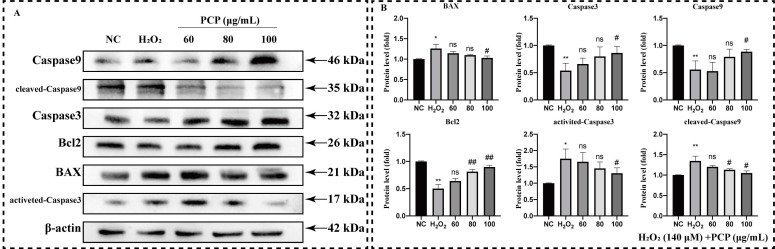
PCP activated BAX/Bcl2/cleaved Caspase3 pathway inside microglia. The cells were treated using H_2_O_2_ (140 μmol) and PCP at different concentrations (0, 60, 80, and 100 μg/mL) for 24 h. The NC group treated with DMEM medium was set up as the control. Then, the levels of BAX, Bcl2, Caspase3, activated Caspase3, Caspase9, and cleaved Caspase9 proteins were evaluated by Western blotting, measuring the grey value using ImageJ. BAX, Bcl2, Caspase3, activated Caspase3, Caspase9, and cleaved Caspase9 protein (**A**) levels. Statistics of the protein’s grey value after β-actin standardization. (**B**) (standardized with β-actin). (^ns^ *p* > 0.05, * *p* < 0.05, ** *p* < 0.01, ^#^ *p* < 0.05, and ^##^ *p* < 0.01).

We first measured the protein level of the BAX/BCL2/cleaved-Caspase3 antiapoptotic pathway in microglia to reveal the mechanism of PCP inhibiting apoptosis (Figure 4A). We observed that H_2_O_2_ treatment significantly elevated the protein levels of BAX, activated Caspase3, and cleaved Caspase9 (*p* < 0.05, Figure 4B). In contrast, the total protein expression levels of Caspase3 and Caspase9 were significantly inhibited (*p* < 0.05, Figure 4B). We also studied the protein levels of the Caspase and BAX families in microglia after H_2_O_2_ and PCP treatment. The results revealed that the total protein levels of Caspase3 and Caspase9 enhanced significantly (*p* < 0.05, Figure 4B). The levels of activated Caspase3 and cleaved Caspase9 decreased significantly (*p* < 0.05, Figure 4B) post-PCP treatment. Moreover, PCP intervention significantly inhibited the expression of BAX, significantly inhibited the expression of BAX, and significantly activated Bcl2 expression (*p* < 0.05, Figure 4B). 

### 2.4. PCP Scavenged H_2_O_2_ Induced Intracellular ROS in Microglia

**Figure 5:**
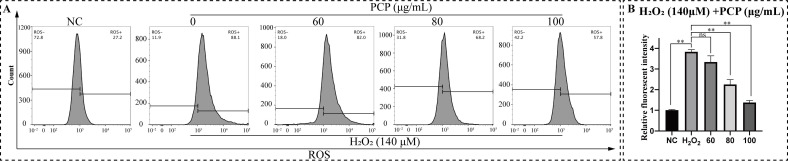
PCP scavenged H_2_O_2_-induced intracellular ROS in the microglia. The cells were treated with H_2_O_2_ (140 μmol) and PCP at different concentrations (0, 60, 80, and 100 μg/mL) for 24 h. The NC group was set as the control and treated with DMEM medium. Then, we stained the cells using a DCFH-DA probe, detected them through a flow cytometer, and established the fluorescence intensity using FlowJo. ROS level in the microglia (**A**). The relative fluorescence intensity of ROS in the microglia (**B**). (^ns^ *p* > 0.05, and ** *p* < 0.01).

We further detected intracellular ROS levels by flow cytometry using a DCFH-DA fluorescent probe to investigate the mechanism of PCP reducing H_2_O_2_-induced cytotoxicity and apoptosis. The treatment method was the same as above, and the relative fluorescent intensity was determined to represent the ROS level (Figure 5A). As observed in microglia, H_2_O_2_ treatment effectively elevated ROS (*p* < 0.05, Figure 5B). However, PCP significantly scavenged intracellular ROS within the treatment group (*p* < 0.05, Figure 5B).

### 2.5. PCP Upregulated NRF2/HO-1 Antioxidant Pathway in Microglia

**Figure 6:**
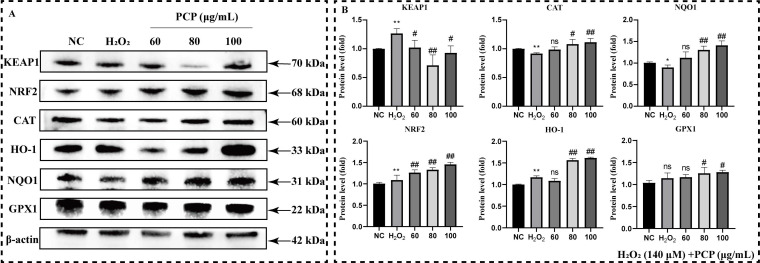
PCP upregulates NRF2/HO-1 antioxidant pathway in microglia. The cells were treated with H_2_O_2_ (140 μmol) and PCP at varying concentrations (0, 60, 80, and 100 μg/mL) for 24 h. The NC group that set and treated with DMEM as the control. Then, we measured the levels of KEAP1, NRF2, CAT, HO-1, NQO1, and GPX1 proteins using Western blotting and estimated the grey value by ImageJ. KEAP1, NRF2, CAT, HO-1, NQO1, and GPX1 proteins (**A**); Statistics of a protein’s grey value after β-actin standardization. (**B**). (^ns^ *p* > 0.05, * *p* < 0.05, ** *p* < 0.01, ^#^ *p* < 0.05, and ^##^ *p* < 0.01).

We measured the protein level of the NRF2/HO-1 antioxidant pathway in microglia to reveal the mechanism of PCP scavenging ROS (Figure 6A). We found that treatment with H_2_O_2_ alone not only significantly elevated the NRF2, KEAP1, and HO-1 protein levels (*p* < 0.05) but also significantly inhibited the CAT and NQO1 protein levels (*p* < 0.05, Figure 6B). The level of GPX1 protein did not enhance significantly (*p* > 0.05, Figure 6B). After PCP treatment, the expression of KEAP1 protein was inhibited significantly (*p* < 0.05, Figure 6B). In addition, the levels of the other five proteins were enhanced (*p* < 0.05, Figure 6B).

### 2.6. PCP Upregulated Ferroptosis Pathway in Microglia

**Figure 7:**
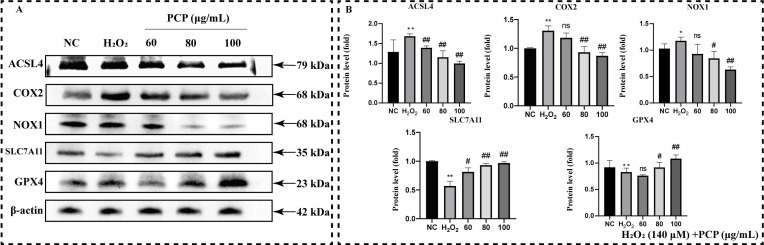
PCP upregulates ferroptosis pathway in microglia. The cells were treated with H_2_O_2_ (140 μmol) and PCP at different concentrations (0, 60, 80, and 100 μg/mL) for 24 h. We set the NC group treated with DMEM medium as the control. Then, the levels of the ACSL4, COX2, NOX1, SLC7A11, and GPX4 proteins were measured by Western blotting, and the grey value by ImageJ. The levels of the ACSL4, COX2, NOX1, SLC7A11, and GPX4 proteins (**A**). The statistics of the proteins’ grey value after β-actin standardization (**B**). (^ns^ *p* > 0.05, * *p* < 0.05, ** *p* < 0.01, ^#^ *p* < 0.05, and ^##^ *p* < 0.01).

We continued to measure the protein level in the ferroptosis pathway that may be activated by the NRF2/HO-1 antioxidant pathway in microglia to reveal the mechanism of PCP intervention in microglia programmed death (Figure 7A). Treatment with H_2_O_2_ alone not only significantly enhanced the ACSL4, COX2, and NOX1 protein levels (*p* < 0.05, Figure 7B) but also inhibited the SLC7A11 and GPX4 protein levels (*p* < 0.05, Figure 7B). After PCP treatment, the SLC7A11 and GPX4 protein levels elevated significantly. The expression of ACSL4, COX2, and NOX1 was all inhibited.

### 2.7. PCP Attenuated ERASTIN/RSL3-Induced Cellular Ferroptosis in Microglia

**Figure 8:**
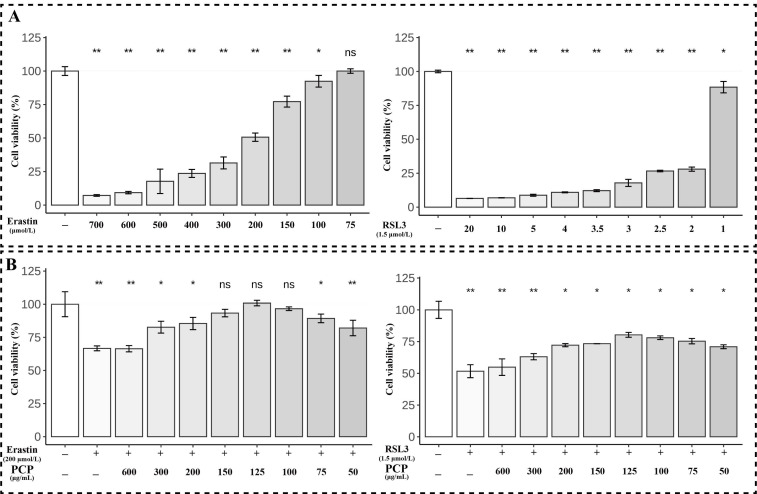
The cell viability in the microglia. The cells were treated using different concentrations of ERASTIN (0, 75, 100, 150, 200, 300, 400, 500, 600, and 700 μmol) or RSL3 (0, 1, 2, 2.5, 3, 3.5, 4, 5, 10, and 20 μmol) for 24 h, then treated with ERASTIN (200 μmol)/RSL3 (1.5 μmol) and PCP at different concentrations (0, 40, 60, 80, 100, 120, 150, 200, and 300 μg/mL), determined by the CCK8 assay. The cell viability of microglia after using ERASTIN, the RSL3 treatment (**A**). The cell viability of microglia after using ERASTIN/RSL3 + PCP treatment (**B**). (^ns^ *p* > 0.05, * *p* < 0.05 and ** *p* < 0.01).

We used two ferroptosis inducers, ERASTIN and RSL3, to simulate the ferroptosis of microglia. In this way, it could be observed how PCP inhibits the ferroptosis of microglia (Figure 8A). The final condition of the simulated ferroptosis of microglia was that the concentration of ERASTIN was 200 μmol or RSL3 was 1.5 μmol, and the intervention time was 24 h. We used different concentrations (60, 80, and 100 μg/mL) of PCP and ERASTIN/RSL3 treated microglia for 24 h and then observed the CCK8 analysis to study the effect of PCP on ERASTIN/RSL3-induced ferroptosis. The CCK8 assay revealed that microglia cell viability was reduced after ERASTIN/RSL3 treatment. However, this harmful effect was enhanced significantly in the PCP-treated groups (*p* < 0.05, Figure 8B).

### 2.8. PCP Scavenged ERASTIN/RSL3 Induced Intracellular ROS in Microglia

**Figure 9:**
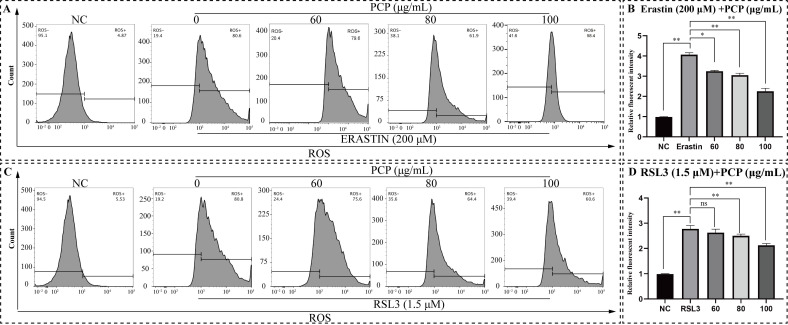
PCP scavenged ERASTIN/RSL3-induced intracellular ROS in the microglia. The cells were treated with ERASTIN (200 μmol) or RSL3 (1.5 μmol) and PCP at different concentrations (0, 60, 80, and 100 μg/mL) for 24 h. We set the NC group treated with DMEM as the control. Then, we stained the cells using a DCFH-DA probe, detected them with a flow cytometer, and established the fluorescence intensity with FlowJo. The ROS level in microglia (**A**,**C**). The relative fluorescence intensity of ROS inside microglia (**B**,**D**). (^ns^ *p* > 0.05, * *p* < 0.05 and ** *p* < 0.01).

By detecting intracellular ROS levels using the DCFH-DA fluorescent probe, both ERASTIN and RSL3 treatment increased the ROS levels in microglia, which was supported by the results from flow cytometry (Figure 9A,C). The treatment method was the same as above, and the relative fluorescent intensity was measured to represent the ROS level (Figure 9A,C). As we observed in microglia, the ERASTIN/RSL3 treatment effectively elevated ROS (*p* < 0.05, Figure 9B,D), but PCP significantly scavenged intracellular ROS within the treatment group (*p* < 0.05, Figure 9B,D).

### 2.9. PCP Reduced ERASTIN/RSL3 Induced Intracellular Fe^2+^ in Microglia

**Figure 10:**
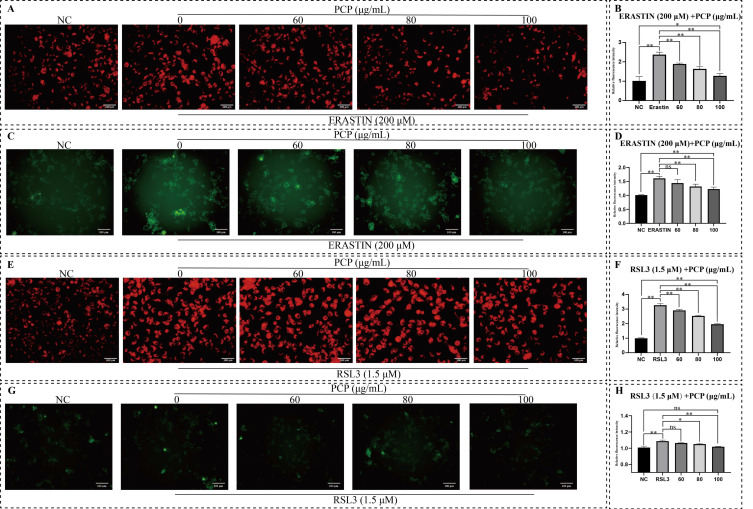
PCP scavenged ERASTIN/RSL3-induced intracellular Fe^2+^ in the microglia. The cells were treated using ERASTIN (200 μmol) or RSL3 (1.5 μmol) and PCP at different concentrations (0, 60, 80, and 100 μg/mL) for 24 h. The NC group was set as the control and treated with DMEM. Then, we stained the cells with a FerroOrange and Mito-FerroGreen probe and observed them under a fluorescent microscope and validated the fluorescence intensity using ImageJ. Intracellular Fe^2+^ level in the microglia (**A**,**E**); Fe^2+^ level in nuclear of the microglia (**C**,**G**). The relative fluorescence intensity of Fe^2+^ level in the microglia (**B**,**D**,**F**,**H**). (^ns^ *p* > 0.05, * *p* < 0.05 and ** *p* < 0.01).

The level of intracellular Fe^2+^ was closely associated with ferroptosis. We used FerroOrange and Mito-FerroGreen to characterize the level of Fe^2+^ in the cytoplasm and nucleus to understand how PCP alleviates ferroptosis induced by ERASTIN or RSL3. It can be observed that the concentration of Fe^2+^ in the cytoplasm and mitochondria was upregulated under the treatment of ERASTIN or RSL3 (Figure 10A,C,E,G). Notably, the concentration of intracellular Fe2^+^ was higher in the case of RSL3 treatment than in the ERASTIN treatment (Figure 10A,E). However, in the mitochondria, the change within Fe^2+^ concentration was significantly smaller after RSL3 treatment than after ERASTIN treatment (Figure 10C,G). This may be due to the more significant effect of RSL3 on cellular iron transport and storage but the smaller impact of RSL3-induced ferroptosis on the Fe^2+^ concentration in mitochondria. Simultaneously, PCP treatment significantly reduced the high Fe^2+^ concentration environment in microglia due to ERASTIN or RSL3 (*p* < 0.05, Figure 10B,D,F,H).

### 2.10. PCP Upregulated Ferroptosis Pathway in Microglia

**Figure 11:**
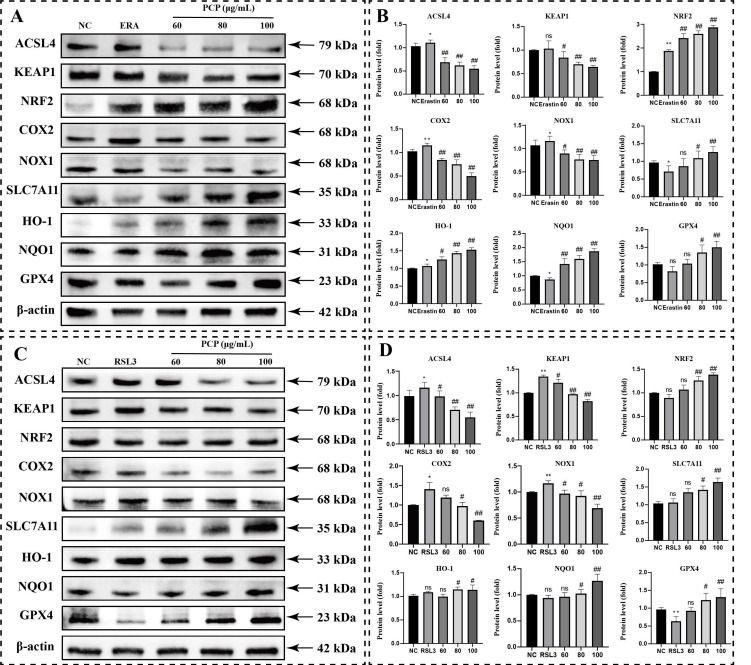
PCP upregulates the ferroptosis pathway in microglia. The cells were treated using ERASTIN (200 μmol) or RSL3 (1.5 μmol) and PCP at different concentrations (0, 60, 80, and 100 μg/mL) for 24 h. The NC group was set up as the control and treated with DMEM. Then, we measured the levels of the KEAP1, NRF2, ACSL4, COX2, NOX1, SLC7A11, HO-1, NQO1, and GPX4 proteins by Western blotting and measured the grey value using ImageJ. The KEAP1, NRF2, ACSL4, COX2, NOX1, SLC7A11, HO-1, NQO1, and GPX4 proteins after ERASTIN/RSL3 intervention (**A**,**C**). Statistics of protein’s grey value after β-actin standardization (**B**,**D**) (standardized with β-actin). (^ns^ *p* > 0.05, * *p* < 0.05, ** *p* < 0.01, ^#^ *p* < 0.05, and ^##^ *p* < 0.01).

We further measured the protein level in the ferroptosis pathway and NRF2/HO-1 antioxidant pathway in microglia to reveal the mechanism of PCP intervention in microglia ferroptosis. We observed that treatment with ERASTIN or RSL3 alone significantly increased the ACSL4, COX2, HO-1, and NOX1 protein levels (Figure 11B,D; *p* < 0.05) but also inhibited the NQO1 protein levels (Figure 11B,D; *p* < 0.05). Notably, after ERASTIN intervention in microglia, the protein expression of SLC7A11 was inhibited, and the protein expression of GPX4 was reduced. In contrast, after RSL3 intervention, the protein expression of GPX4 was significantly provided, while the protein expression of SLC7A11 was not affected. The protein expression levels of NRF2 and KEAP1 did not change significantly after treatment using ERASTIN or RSL3 (Figure 11B,D; *p* > 0.05). After PCP treatment, the NRF2, HO-1, SLC7A11, and GPX4 protein levels enhanced significantly. The expression of ACSL4, KEAP1, COX2, and NOX1 was all inhibited.

## 3. Discussion

As macromolecular aggregates, plant polysaccharides cannot directly penetrate the cell membranes but initiate relevant immune regulation and response by binding to receptors on the cell membrane [33]. These are pattern recognition receptors (PRR), including the Toll-like receptor family, mannose receptor family, and C-type lectin receptors [34,35]. It is reported that only TLR2 and TLR4 in the human body can recognize polysaccharides, among which P polysaccharides could enter cells and mediate MAPK signaling through the TLR4 receptors [36,37]. Thus, PCP could enter microglia through TLR4 and regulate the MAPK signaling pathway. However, further studies are required to validate this hypothesis.

Several studies report that herbal polysaccharides could treat cancer by increasing ROS levels in tumor cells [38]. Moreover, it can mitigate nerve cell death by reducing oxidative stress [39]. The present study confirmed the role of PCP in alleviating microglia RCD by activating the NRF2/HO-1 signaling pathway. PCP is also a traditional herbal extract, and the polysaccharides exhibit opposite effects in different cells could be related to structural differences between PCP and other polysaccharides or the differential internal environment of different cells and receptor properties [40,41]. The biological function of a polysaccharide is determined by the type of monosaccharide it is composed of, the monosaccharide ratio, the glycosidic bond, the side chain structure, and the molecular weight [42,43]. The PCP obtained from our study was subjected to infrared spectroscopy, molecular mass analysis, and monosaccharide composition analysis. Several studies have reported the structure of PCP and elucidated its effect on physiological functions. Therefore, more studies are required on the stereological structure and target sites of PCP to confirm the hypothesis.

Neuroglia is crucial in neurodegenerative diseases, with microglia essential in maintaining homeostasis and regulating neuronal surveillance inside the brain [44,45]. The cell numbers of microglia reduce, and their functions weaken, leading to the loss of their neuroprotective capacity. This loss of capacity largely contributes to the development of the disease. Aging predisposes microglia to exposure to an unfavorable peroxidative environment, which causes elevated ROS in this case [46,47]. It was also revealed that neuronal cells, including microglia, can maintain relative cellular stability within a certain level of ROS, and its overload leads to the programmed cell death of microglia [48]. In neurodegenerative diseases, the unbalanced antioxidant systems and ROS overload beyond the regulatory capacity of cells could damage microglia and lead to reduced viability and differentiation [49]. In the present study, different concentrations of H_2_O_2_ were used to simulate ROS overload in cells. When microglia were treated with H_2_O_2_, their survival rate reduced, and their apoptosis rate enhanced. This trend could be alleviated by cotreatment with PCP, and it alleviated the ROS overload triggered through H_2_O_2_ intervention. Studies have shown that activating the NRF2/ARE antioxidant pathway is vital to scavenge reactive oxygen species in neuronal cells. In our experiments, the protein levels of NRF2 and HO-1 were upregulated within cells after the H_2_O_2_ treatment of microglia alone. The protein ratio of BAX to Bcl2 was simultaneously upregulated, and Caspase3 protein and Caspase9 protein were activated. This implies that H_2_O_2_-induced oxidative stress started the NRF2/HO-1 pathway but was insufficient to maintain redox homeostasis causing microglial cell apoptosis. Meanwhile, alterations in various ferroptosis-related proteins, including ACSL4, COX2, SLC7A11, and GPX4, depict that oxidative stress could simultaneously lead to multiple programmed deaths, including ferroptosis in microglia. However, PCP treatment elevated the NRF2/HO-1 antioxidant pathway and restored the balance of apoptosis and ferroptosis-related pathways. Therefore, PCP could protect microglia from oxidative stress damage by activating the NRF2/HO-1 antioxidant pathway and scavenging for reactive oxygen species.

From another point of view, aging also causes the accumulation of ferrous ions in microglia. Under normal conditions, the iron uptake of cells and storage capacity are in dynamic equilibrium. Iron levels increase when this is out of balance, and the susceptibility to ferroptosis increases [50]. After the accumulation of iron-dependent ROS triggered through the upstream pathway, depletion of polyunsaturated fatty acid phospholipids (PUFA-PLs) will be induced, leading to the development of ferroptosis. Simultaneously, inhibition of Acyl–CoA synthetase long-chain family member4 (ACSL4) can enhance resistance to ferroptosis [51,52,53,54]. In the present study, we used different concentrations of ERASTIN or RSL3 to simulate cellular ferroptosis. Their survival was significantly reduced when microglia were treated with ERASTIN/RSL3. Treatment with PCP alleviated this trend while scavenging intracellular ROS and reducing the Fe^2+^ concentrations. GPX4 and SLC7A11 have been reported to be critical proteins for cellular resistance to ferroptosis. In this experiment, not only was GPX4 protein inhibited by RSL3, but ERASTIN inhibited SLC7A11 after treatment using ferroptosis inducers without PCP. Other ferroptosis-related proteins, such as ACLS4, NQO1, COX2, and NOX1, revealed specific expression upon ferroptosis [55]. It was demonstrated that NOX1 reacts with NADPH and reduces its level after the expression of PTGS2 (COX2) is activated, leading to the accumulation of ROS and thus triggering ferroptosis. This implies that the inducer in this experiment caused an imbalance of intracellular ROS and Fe^2+^ levels, leading to cellular damage in microglia. However, the treatment of PCP enhanced the NRF2/HO-1 pathway and restored the balance of the associated downstream proteins. Therefore, we suggest that PCP can protect microglia from cellular damage due to ferroptosis by activating the NRF2/HO-1 pathway.

Additionally, PCP treatment alone did not affect ROS levels or the NRF2/HO-1 pathway in microglia. This result indicates that PCP can affect redox homeostasis during oxidative stress or ferroptosis state but does not affect the normal physiological function of microglia under normal circumstances. This suggests that the microglial cell state influences the action of PCP. Under normal conditions, it does not impact the antioxidant system and other systems that control programmed cell death. However, it can restore intracellular homeostasis in states induced by oxidative stress or ferroptosis. This maintains endostasis in the nervous system.

Therefore, PCP can improve microglia survival by activating the NRF2/HO-1 pathway and scavenging intracellular ROS levels under oxidative stress conditions and Fe^2+^ levels under ferroptosis-inducing conditions. Our findings are relevant, because they depict that PCP not only acts on apoptosis due to oxidative stress in the nervous system but also protects neuronal cells from the effects of ferroptosis. This provides novel ideas for the clinically effective prevention and treatment of diseases due to the accumulated reactive oxygen species in the CNS. Simultaneously, it has been established that PCP has the development potential as a new drug candidate in treating neurodegenerative diseases.

## 4. Material and Method

### 4.1. Reagents

Ethanol, Chloroform, Methanol, α- Naphthol, Trifluoroacetic acid, Sodium hydroxide, Sodium acetate, Hydrogen peroxide (H_2_O_2_), and 2′7′-Dichlorofluorescin diacetate were purchased from Sigma-Aldrich (St. Louis, MO, USA). Fucose, Rhamnose, Arabinose, Galactose, Glucose, Xylose, Mannose, Fructose, Ribose, Glucuronic acid, Mannuronic acid, Guluronic acid, and Galacturonic acid were purchased from National Institutes for Food and Drug Control. ERASTIN and RSL3 were purchased from Selleck. The annexin V-FITC Apoptosis Detection Kit was purchased from MultiSciences (LiankeBio). The Hoechst 33342/PI Double Stain Kit was purchased from Slarbio. Dulbecco’s modified Eagle’s medium (DMEM) was purchased from Procell. The Cell Counting Kit-8 (CCK8) assay was purchased from Vazyme. Fetal bovine serum (FBS) was purchased from Gibco. Primary antibodies form β-actin, Caspase3, Cleaved Caspase9, Caspase9, NRF2, KEAP1, HO-1, GPX4, ACSL4, and NQO1 were purchased from Abcam. Primary antibody for BAX, Bcl2, SLC7A11, GPX1, and CAT from Proteintech Group. FerroOrange and Mito-FerroGreen were purchased from Dojindo Laboratories.

### 4.2. Preparation and Purification of Polysaccharides

The herb used in this project was identified by Liu Hao, a researcher at the Institute of Traditional Chinese Medicine, Hunan Provincial Institute of Traditional Chinese Medicine, as the rhizome part of the plant *Polygonatum cyrtonema* Hua. The crude polysaccharide of *Polygonatum* polysaccharides was extracted using water extraction and alcohol precipitation. The ratio of solid to liquid was 20:1, the extraction temperature was 80 °C, the time was 2.5 h, and the extraction was performed twice. The extracts were combined, the solution was centrifuged, and the supernatant was obtained. We used Savage reagent (5:1 ratio of chloroform:n-butanol) to remove protein from the crude polysaccharide. Decolorization was subsequently performed through a macroporous adsorption resin. Elution was carried out with 30% ethanol solution, the flow rate was 2 mL/min, the ratio of the loading extract, and D101-macroporous adsorption resin was 1:15. We evaporated the purified polysaccharides under reduced pressure to an extracted state. Then, we used freeze-drying under reduced pressure to dry the polysaccharides.

### 4.3. Total Sugar Content and Functional Groups of Polysaccharides

The dried polysaccharide powder was taken into an agate mortar, and KI powder was added as a dispersant and ground evenly. An appropriate uniform fine powder was obtained and spread on the mold, pressed at a pressure of 20 MPa for 1 min, the light was checked to confirm that the sample was uniform, and the plate was light-transmitting. The test sample is ready for consumption. The prepared PCP samples were placed inside a Fourier-transform infrared spectrometer (Thermo Scientific Nicolet iS10) for scanning; the spectral scanning range was 4000–400 cm^−1^, with 32 scans performed, and the resolution was set to 4 cm^−1^. The background of H_2_O and CO_2_ should be deducted during the scanning.

### 4.4. The Molecular Weight of Polysaccharides

The samples were dissolved in 0.1 M NaNO_3_ aqueous solution containing 0.02% NaNO_3_ at 1 mg/mL concentration and filtered through a 0.45 μm pore size filter. The homogeneity and molecular weight of various fractions were determined using SEC-MALLS-RI. The weight- and number-average molecular weight (Mw and Mn) and polydispersity index (Mw/Mn) of various fractions within 0.1 M NaNO_3_ aqueous solution containing 0.02% NaNO3 were evaluated using a DAWN HELEOS-II laser photometer (He-Ne laser, λ = 663.7 nm, Wyatt Technology Co., Santa Barbara, CA, USA) equipped with three tandem columns (300 × 8 mm, Shodex OH-Pak SB-805, 804, and 803; Showa Denko KK, Tokyo, Japan), held at 45 °C using a model column heater. The flow rate was 0.4 mL/min. A differential refractive index detector (Optilab T-rEX, Wyatt Technology Co., Santa Barbara, CA, USA) was simultaneously processed to give the concentration of fractions and the dn/dc value. The dn/dc value of the particles within 0.1 M NaNO_3_ aqueous solution containing 0.02% NaNO_3_ was determined 0.141 mL/g. The data were acquired and processed with ASTRA6.1 (Wyatt Technology). The quantified data were fed into an Excel format.

### 4.5. Monosaccharide Composition of Polysaccharides

Approximately 5 mg of the sample was hydrolyzed using trifluoroacetic acid (2 M) at 121 °C for 2 h in a sealed tube, and the sample was dried with nitrogen. Methanol was added to wash, blow dry, and repeat methanol wash 2–3 times. For evaluation, the residue was redissolved in deionized water and filtered through 0.22 μm microporous filtering film. The sample extracts were analyzed using high-performance anion-exchange chromatography (HPAEC) over a CarboPac PA-20 anion-exchange column (3 by 150 mm; Dionex) through a pulsed amperometric detector (PAD; Dionex ICS 5000 system). Flow rate, 0.5 mL/min; injection volume, 5 μL; solvent system, B: (0.1 M NaOH, 0.2 M NaAc); gradient program, 95:5 *v*/*v* at 0 min, 80:20 *v*/*v* at 30 min, 60:40 *v*/*v* at 30.1 min, 60:40 *v*/*v* at 45 min, 95:5 *v*/*v* at 45.1 min, and 95:5 *v*/*v* at 60 min. The data were acquired on the ICS5000 (Thermo Scientific, Waltham, MA, USA) and processed using chameleon 7.2 CDS (Thermo Scientific, Waltham, MA, USA). The quantified data were output into the Excel format.

### 4.6. Cell Culture

Microglia were procured from the Wuhan Proceeds Company. Mouse microglia BV2 Cells were cultured in DMEM supplemented using 10% FBS (Gibco; Carlsbad, CA, USA) and 1% penicillin–streptomycin antibiotic mix. Cells were kept at 37 °C in a humidified incubator with 5% CO_2_. The microglia between passages 4 and 10 were subsequently used in experiments.

### 4.7. Cell Viability

Cells were grown in 96-well plates at a density of 3 × 10^3^ cells/well and preserved in the medium for 24 h. The cells were treated using different concentrations of H_2_O_2_ (0, 40, 60, 80, 100, 120, 140, 160, 180, and 200 μmol), ERASTIN (Ferroptosis Inducer by Suppressing SLC7A11 Protein Expression) (0, 75, 100, 150, 200, 300, 400, 500, 600, and 700 μmol) or RSL3 (Ferroptosis Inducer by Suppressing GPX4 Protein Expression) (0, 1, 2, 2.5, 3, 3.5, 4, 5, 10, and 20 μmol) for 24 h. Cells were then incubated for an additional 24 h to avoid. The appropriate concentration of H_2_O_2_, ERASTIN, or RSL3 was chosen, and different concentrations of PCP (0.125, 0.25, 0.5, and 1 mg/mL) were used to prevent cell damage. Before measurement, 20 μL of CCK8 was added to each well and incubated for 4 h at 37 °C. A multimode plate reader measured the absorbance value at 450 nm (PerkinElmer, Waltham, MA, USA).

### 4.8. Hoechst 33342/PI Double Fluorescence Staining

The microglia were seeded in a 12-well plate at a density of 5 × 10^4^ cells/well, and the cells were incubated for 24 h. Each group of cells was treated using the appropriate H_2_O_2_, ERASTIN, or RSL3, with different PCP concentrations (0.125, 0.25, 0.5, and 1 mg/mL) for 24 h. The cell culture medium was discarded and washed twice using PBS. Next, a complete medium with 1% Hoechst 33342 (100×) and 1% PI was introduced to each well and incubated for 15 min. After that, the dye was discarded and washed with PBS 2–3 times. After adding 500 μL PBS to each well, the cells were placed in the dark under an inverted fluorescent microscope to measure the fluorescence intensity and determine the apoptosis level.

### 4.9. Apoptosis Detection Assay

The microglia were seeded in 6-well plates at a density of 1 × 10^5^ cells/well. The cells in each group were treated with 140 μmol/mL H_2_O_2_ and different concentrations (0, 60, 80, and 100 μg/mL) of PCP for 24 h. A small amount (0.25%) of trypsin was added to digest the cells; after which, the cells were washed twice using PBS. Next, the Annexin V-FITC/PI Apoptosis Kit was used to load fluorescent probes. A 100 μL 1× working solution to resuspend cells, 5 μL Annexin V-FITC, and 10 μL PI reagent to each group, mix well and avoid incubating in light for 10 min at room temperature. After the probes were started, Annexin V-FITC/PI double fluorescence staining was utilized to detect the apoptosis level using flow cytometry.

### 4.10. Detection of Intracellular ROS

The cells were cultured in 6-well plates. After the treatment, cells were washed twice with PBS solution. The DCFH-DA probe was diluted to a concentration of 1:1000 in DMEM medium, added to each well, and cultured for 4 h. It was kept in an ultra-high-speed flow cytometer for detection to obtain the intracellular reactive oxygen species level, processed with FlowJo 10.0 software for the overall statistical analysis.

### 4.11. Detection of Intracellular Fe^2+^

The cells were cultured in 6-well plates. After the treatment, the cells were washed three times with serum-free medium; added FerroOrange or Mito-FerroGreen working solution with a concentration of 1 μmol/L; and incubated in a carbon dioxide incubator for 30 min, taken out, and placed in a fluorescent inversion in the dark. Photographs were captured using a microscope (FerroOrange: excitation light at 543 nm and emission light at 580 nm. Mito-FerroGreen: excitation light at 505 nm; emission light at 535 nm). ImageJ 8.0 software analyzed the fluorescence intensity of cells, and statistical analysis was undergone to determine the intracellular Fe^2+^ concentration.

### 4.12. Protein Extraction and Western Blotting

The cells were cultured in 6-well plates. After treatment, cells were digested using trypsin and collected in 1.5 mL tubes. We washed cells twice using PBS and added 500 μL of RIPA lysis buffer (Thermo Fisher, Waltham, MA, USA). Based on the manufacturer’s protocol, we placed the tube on ice for 30 min and shook it every 5 min to completely lyse the cells. The cells were centrifuged (200× *g*, 4 °C, 15 min), transferred the supernatant to a new tube, and measured the total protein concentration with the BCA protein assay kit (Biosharp). The protein with 5× loading buffer (Thermo Fisher Scientific, Waltham, MA, USA) was boiled at 100 °C for 10 min and then stored at −80 °C.

Western blots were undergone using polyacrylamide gel electrophoresis (PAGE), and 15 micrograms of protein in each group were separated and transferred to the PVDF. Blocked for one hour by using 5% skim milk powder solution. After antigen blocking, PVDF was incubated in 1:1000 dilution of primary antibody at 4 °C for 16 h, then utilized TBST to clear the membrane and incubated at 1:10,000 dilution of chemiluminescent secondary antibody at 37 °C for 1 h. The imaging system was used to detect luminescence intensity.

### 4.13. Statistics and Analysis

Data in this study were expressed as the mean ± standard deviation (SD) and were statistically analyzed using GraphPad Prism (version 8.0) or SPSS (version 23.0), and multiple comparisons were performed using the Student’s *t*-test or one-way analysis of variance (ANOVA). Grayscale values of WB protein bands were normalized with β-actin protein. Values of *p* < 0.05 were considered to be significantly different. All experiments were repeated at least three times. In the statistical results of WB protein bands, * and ** are the statistical results of NC compared with the model group, and # and ## are the statistical results of the model group compared with the PCP intervention group.

## 5. Conclusions

In this study, the role of PCP in microglia was analyzed, and it was observed for the first time that PCP could resist microglial ferroptosis by activating the NRF2/HO-1 antioxidant pathway. The results revealed that NRF2/HO-1 pathway was activated under H_2_O_2_ treatment. We further studied the role of PCP in oxidative stress and showed that PCP could activate the BAX/Bcl2/cleaved caspase3 antiapoptotic pathway and reduce H_2_O_2_-induced microglia toxicity and apoptosis. Simultaneously, the PCP-activated NRF2/HO-1 antioxidant pathway cleared intracellular ROS under oxidative stress. Under the intervention of ferroptosis inducers such as ERASTIN and RSL3, the Fe^2+^ concentration in microglia increased, and ferroptosis occurred. PCP activates the SLC7A11/GSH/GPX4 pathway of microglia by upregulating the NRF2 expression and reducing the concentration of ROS and Fe^2+^ in the microglia.

## Data Availability

This study did not report any data.

## Sample Availability

Not available. Samples of the compounds are not available from the authors.
